# Understanding the role of Toll-like receptors in lung cancer immunity and immunotherapy

**DOI:** 10.3389/fimmu.2022.1033483

**Published:** 2022-10-31

**Authors:** Bettina Hoden, David DeRubeis, Margarita Martinez-Moczygemba, Kenneth S. Ramos, Dekai Zhang

**Affiliations:** ^1^ Center for Infectious and Inflammatory Diseases, Institute of Biosciences and Technology, Texas A&M University, Houston, TX, United States; ^2^ Center for Genomic and Precision Medicine, Institute of Biosciences and Technology, Texas A&M University, Houston, TX, United States

**Keywords:** Toll-like receptors, lung cancer, innate immunity, cancer immunity, immunotherapy, immune checkpoint inhibitor

## Abstract

Lung cancer is currently the leading cause of cancer-related deaths worldwide. Significant improvements in lung cancer therapeutics have relied on a better understanding of lung cancer immunity and the development of novel immunotherapies, as best exemplified by the introduction of PD-1/PD-L1-based therapies. However, this improvement is limited to lung cancer patients who respond to anti-PD-1 immunotherapy. Further improvements in immunotherapy may benefit from a better understanding of innate immune response mechanisms in the lung. Toll-like receptors (TLRs) are a key component of the innate immune response and mediate the early recognition of pathogen-associated molecular patterns (PAMPs) and damage-associated molecular patterns (DAMPs). TLR signaling modulates the tumor microenvironment from “cold” to “hot” leading to immune sensitization of tumor cells to treatments and improved patient prognosis. In addition, TLR signaling activates the adaptive immune response to improve the response to cancer immunotherapy through the regulation of anti-tumor T cell activity. This review will highlight recent progress in our understanding of the role of TLRs in lung cancer immunity and immunotherapy.

## Introduction

Toll-like receptors (TLRs) recognize both pathogen-associated molecular patterns (PAMPs) and endogenous damage-associated molecular patterns (DAMPs). Ample evidence has demonstrated that TLRs play a critical role in cancer development and treatment. TLR signaling activation not only triggers innate immune responses but also regulates adaptive immunity responses. As such, TLRs and TLR signaling serve as targets for monitoring cancer progression and the development of a new strategy for cancer treatment. Lung cancer has been the leading cause of cancer-related deaths since the 1950s in men and the 1980s in women, surpassing breast cancer (1). Conventional treatments including surgery, chemotherapy, and radiation therapy have proven to be insufficient for combating the steady increase in mortality rate, especially when patients are diagnosed late, and cancer has invaded other organs. This has led to increasing interest in immunotherapies, particularly the use of immune checkpoint inhibitors like programmed cell death protein (PD-1) and cytotoxic T-lymphocyte-associated antigen 4 (CTLA-4) antibodies (2). However, patients are often found to relapse with checkpoint inhibitors or show resistance to antibody therapy entirely, thus the initial success of immunotherapy is limited to a smaller group of patients (3). Despite these challenges, the positive impact of immunotherapies on lung cancer mortality outcomes provides compelling evidence that targeting immune elements may be a promising approach, especially as the underlying immunologic mechanisms continue to be unraveled. This has promoted an expansion of the field beyond targeting adaptive T cells in current immunotherapies to include innate immunity components like TLRs (4). This review will summarize the roles of TLRs in lung cancer and their current utilization in recent studies, including the use of TLR agonists alone or in combination with immune checkpoint inhibitors, for lung cancer treatment.

## Toll-like receptor synopsis

The host immune response relies on the actions of both innate and adaptive immunity, but the importance of innate immunity was not fully recognized until the discovery of TLRs approximately 25 years ago (5). The innate immune response recognizes pathogens upon invasion of the host through receptors called pattern recognition receptors (PRRs). These PRRs include Toll-like receptors (TLRs), NOD-like receptors (NLRs), RIG-I-like receptors (RLRs), and cytosolic sensors for DNA (6). These receptors recognize different molecular patterns of pathogens known as pathogen-associated molecular patterns (PAMPs) and also recognize endogenous danger signals known as damage-associated molecular patterns (DAMPs) (7). PAMPs can be expressed by pathogens or invasive microbes and DAMPs are stress signals released by damaged cells. Amongst PRRs, TLRs are the first identified and well-studied family of receptors responsible for the initiation of the immune response through the activation of macrophages, maturation of dendritic cells (DCs), and recognition of host vs. non-host antigens that if unchecked, can result in auto-immune disorders (8). TLRs are mainly expressed on innate immune cells such as macrophages, dendritic cells, natural killer cells, and neutrophils; they are also expressed on T and B lymphocytes, epithelial cells, endothelial cells, and fibroblasts (9). Based on their cellular localization, TLRs can be divided into two major categories: 1) those expressed on the cell surface and mainly involved in the recognition of microbial membrane molecular patterns, including TLR1, TLR2, TLR4, TLR5, TLR6, and TLR10; 2) those located intracellularly within endosomes and mainly involved in the recognition of microbial-derived nucleic acids, including TLR3, TLR7, TLR8, and TLR9 (6).

**Figure 1:**
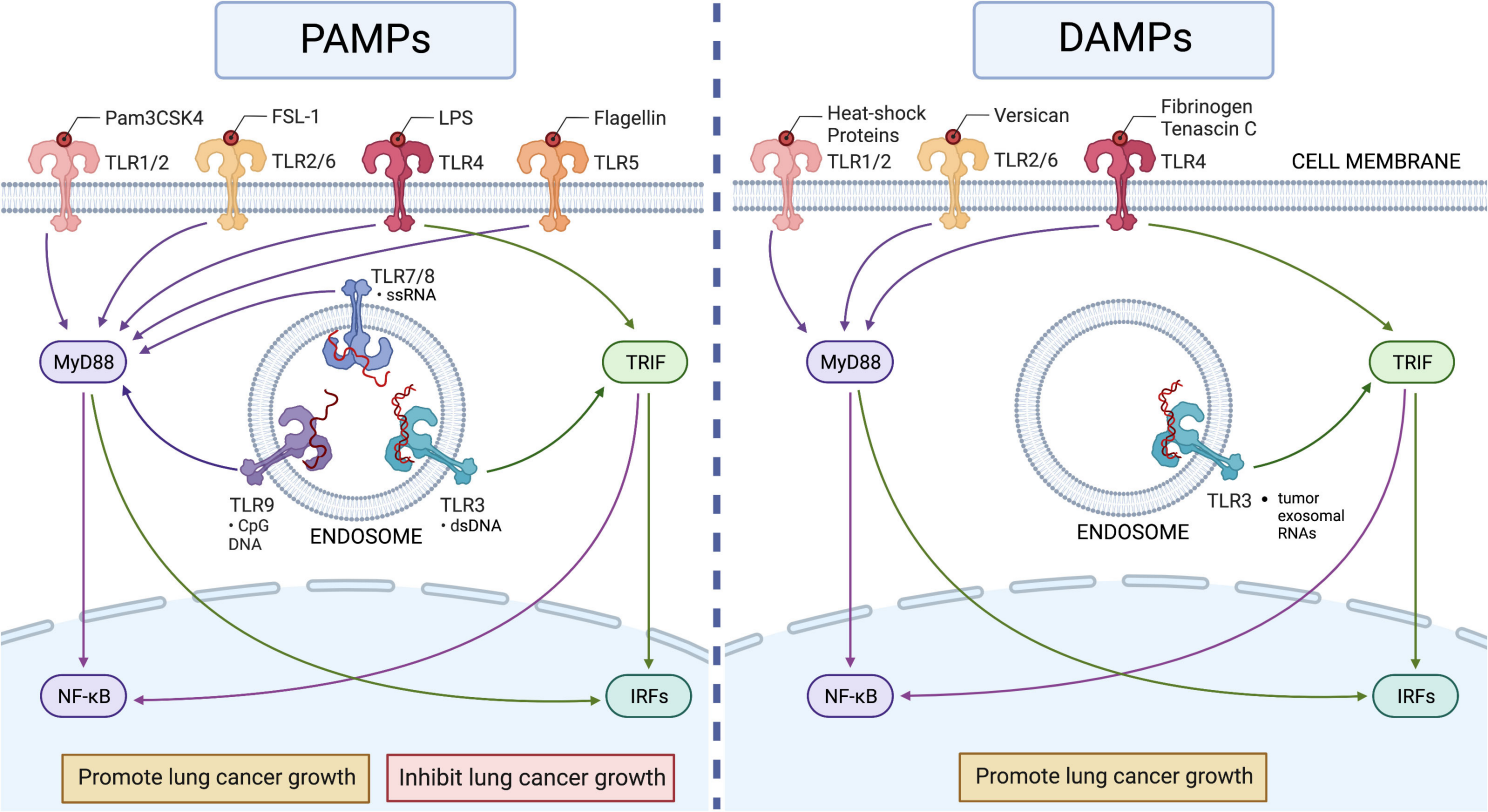
The roles of TLRs in lung cancer. Left panel: Human TLR1 to TLR9 recognize their ligands in pathogens (PAMPs) to activate MyD88-dependent (for all TLRs except TLR3) and TRIF-dependent (only for TLR3 and TLR4) pathways to induce innate immune responses in lung cancer. The activation of TLRs can show both pro- and anti-tumor activities depending on the setting. Right panel: Human TLR2 to TLR4 recognize endogenous ligands (DAMPs) in lung cancer, but these recognitions appear to induce only pro-cancer activity (**Table 1**). This figure was generated using BioRender.

TLRs are type I integral transmembrane proteins, including the leucine-rich repeat (LRR) domain, a transmembrane domain, and the Toll-interleukin receptor (TIR) domain (10). The LRR motifs of TLRs bind to its recognized PAMPs or DAMPs resulting in dimerization and recruitment of the TLR signal transduction TIR domain adaptor proteins (MyD88, TIRAP/MAL, TRIF, TRAM, and SARM) to activate gene expression through MyD88-dependent or -independent pathways (11). In the MyD88 dependent pathway, the MyD88 protein directly interacts with the TLR TIR domain of all TLRs, except TLR3. The formation of this complex activates the NF-κB (nuclear factor kappa light chain enhancer of activated B cells) or AP-1 (activator protein 1) signaling pathway. TLR3 uses the MyD88 independent pathway, also known as the TRIF-dependent pathway, while TLR4 is the only receptor capable of using both pathways. Upon ligand stimulation, either the TRIF or TRAM molecule is recruited to the TLR TIR domain leading to delayed NF-kβ activation downstream. These signals are responsible for the induction of several interferon (IFN) genes (8). Transduction through these pathways is associated with the recruitment of both pro-inflammatory cytokines and co-stimulatory molecules to promote inflammatory responses (**Figure 1**). Besides their traditional role in innate immunity, TLRs also serve as a bridge to adaptive immunity. Without TLR signaling, adaptive immunity is weak and insufficient to support the survival of the host. TLRs aid in the maturation of DCs, which in turn allow the host to have optimal antigen presentation upon pathogen detection. Therefore, the next section will highlight the different roles of TLRs in lung cancer immunity and progression and their role in both innate and adaptive immune alterations.

## Toll-like receptors in lung cancer

### Lung cancer overview

Lung cancer is currently the world’s leading cause of cancer-related deaths (12). These deaths are largely due to asymptomatic progression and escape of immune surveillance until the disease has advanced to a metastatic stage (12, 13). Although lung cancer can affect different age populations, the risk of developing this disease increases after age 50 (12). There are two types of lung cancer: small cell lung cancer (SCLC) and non-small cell lung cancer (NSCLC). SCLC is less common as it originates from the neuroendocrine cells of the lung. NSCLC is the most common consisting of three subtypes: squamous cell carcinoma, large cell carcinoma, and adenocarcinoma – the latter being the most common type. For tumor cells to survive in the host, they must acquire a vascular supply (13). To achieve this, tumor cells must communicate with the surrounding microenvironment to initiate angiogenesis following the release of angiogenic growth factors such as vascular endothelial growth factor (VEGF) and/or through cooperation with the tumor microenvironment (TME) (13). Within the TME, innate immune macrophages are the most common cell type. The role of macrophages depends on their phenotype, M1 or M2, which can either act through inflammatory responses against tumor invasion, or promote tumor progression, respectively (14). Tumor cell initiation, migration, and invasion are associated with the progressive acquisition of genetic mutations. The resulting mutant proteins can be recognized by antigen-presenting cells (APCs) such as macrophages and DCs, as well as lymphocytes that attack the tumor cells (15). However, tumor cells have advanced their ability to modulate the innate immune system by forcing macrophages to differentiate into pro-tumor M2 types or by altering the antigens presented by DCs to afford tumor cell protection. Upon successful clonal expansion of tumor cells, and with provided access to nutrients and immune cell protection, tumor cells can expand uncontrollably and migrate to distant sites (13). Thus, the crosstalk between tumors and immune cells is critical for tumor development. As such, TLRs may serve as a hub for crosstalk during cancer progression.

### The variable roles of TLRs in lung cancer cells and the TME

Vast amounts of evidence have demonstrated that TLRs play an important role in lung cancer development and treatment. However, opposing roles for either pro- or anti-tumor progression have been described for different TLRs (**Figure 1**). TLRs are expressed on both resident lung epithelial cells as well as myeloid, lymphoid, and immune infiltrating cells (16). The ability for TLRs to be activated in both lung cancer epithelial cells and immune cells may contribute to oppositional response profiles (16). Activation of lung epithelial TLRs promotes chemokine production and VEGFs, while activation of innate immune TLRs enhances adaptive antigen processing and T-cell activation (17). The balance between parenchymal and immune cell regulation defines the TME, ultimately dictating the establishment and progression or regression of tumor cells (18).

Lung DCs can be divided into either conventional or plasmacytoid DCs (pDCs) (19). DCs have an essential TLR-dependent response that bridges the innate and adaptive immune system during the progression of lung carcinomas. TLR2 and TLR4 exert Th1- or Th2-like proliferative immune responses in lung-derived DCs, which normally have low activation in the absence of TLRs (20). Lung cancer is considered to have a Th2-like (pro-tumor) microenvironment dominated by immature DCs. pDCs highly express TLR7/8 and TLR9 (21). TLRs that are highly expressed in lung carcinoma cells are presented in **Table 1**.

**Table 1:** Roles of TLRs in lung cancer.

TLR Pathway	Main Cell Types Involved	Pro-tumorigenic Activity	References
**TLR2**	Lewis lung carcinoma MDSCs	TLR2/6 promote tumor growth and metastasis	(22)
	M1 macrophages	TLR1/2 promote M1 macrophages	(23)
**TLR3**	Tumoral exosomes Lung epithelial	Sensing tumoral RNA to promote metastasis	(24, 25)
**TLR4**	Human lung cancer	Upregulated in lung carcinomas, induces immunosuppressive cytokines and resistance to apoptosis	(26)
	NSCLC	Upregulates PD-L1 expression	(27)
**TLR7**	NSCLC	Promotes tumor progression and chemotherapy resistance	(28)
**TLR8**	Primary lung tumors	Upregulated in lung carcinoma	(29)
**TLR9**	Human lung tissue	Upregulated in lung carcinoma	(30)
		Promotes metastasis and progression	(31)
**TLR Pathway**	**Main Cell Types Involved**	**Anti-tumorigenic Activity**	**References**
**TLR2**	Mast cells	TLR2 activation on mast cells reversed pro-tumor role	(32)
**TLR3**	NSCLC	Induces apoptosis Reactivate local innate responses	(33)
**TLR4**	NSCLC DCs	Inhibits NSCLC by regulation	(34, 35)
**TLR5**	NSCLC	Upregulates anti-tumor effect	(36)
**TLR7/8**	DC and NK	Enhances DC and NK cell activation	(37, 38)
**TLR7**	NSCLC	Inhibits angiogenesis	(39)
**TLR9**	PBMCs Human primary	TLR9 activation by CpG-ODN induces an anti-tumor activity	(40)

Under normal conditions, macrophages are seen in high numbers within the lung TME. TLR2, TLR3, TLR4, and TLR6 are expressed at higher levels than TLR7 and TLR9 in lung macrophages (41). Upon ligand stimulation, TLRs can induce both M1 and M2 phenotypes (42). It has been shown that TLR2 and TLR4 activation increases tumor progression by tumor-associated macrophages perhaps due to higher expression of these innate cells (22, 41). It should be noted, however, that TLR9 activation with CpG-ODN type B in a lung tumor mouse model has been associated with a high influx of macrophages to the lung (19).

Natural killer (NK) cells are important innate immune cell effectors used against tumor cells. They are activated by DCs, and their cytotoxicity is induced through the MyD88-independent pathway. Mast cells are another innate immune cell that abundantly surrounds solid tumors and are known to be pro-tumor by enhancing tumor angiogenesis (32). Innate myeloid-derived suppressor cells (MDSCs) negatively regulate the immune system and are important components of the TME (23). Recent studies have shown that TLR signaling can regulate the differentiation and function of MDSCs [27, 28]. **T**he activation of TLR2 signaling in MDSCs induces tumor regression. TLR2 and TLR 7/8 are highly expressed in monocytic-MDSCs (m-MDSCs) making them an important aid for tumor immune evasion (43, 44). Furthermore, agonists targeting TLR 1/2 cause m-MDSCs to mature into M2 immunosuppressive phenotypes while agonists targeting TLR 7/8 cause m-MDSCs to differentiate into M1 (45).

Cytotoxic T lymphocytes are the major adaptive cellular effectors against tumor regression. Tumor regression is dependent on the MyD88-dependent signaling pathway involved in the expression of MHC-I. Humoral immunity encompasses the adaptive immune response that occurs through B lymphocyte antibody production. Normally the B cell numbers in the lung are considerably low, but significantly increased in lung cancer (46). TLR7 and TLR9 are highly expressed in B cells (21). A mouse model of lung carcinoma showed that TLR9 activation in B cells promotes tumor regression (47). TLR9 stimulation with CpG-ODN increased lung metastasis in the absence of IL-17, a proinflammatory cytokine (48).

### TLR2 in lung cancer

TLR2 is known to heterodimerize with its co-receptors: TLR1 or TLR6. These combinations allow for increased diversity in ligand recognition (49). It has been demonstrated that the TLR2:TLR6 complex activates immune responses, while the TLR2:TLR1 complex suppresses T cell immunity (23). The TLR2:TLR6 complex has been linked with enhanced lung cancer metastasis (23). For distant-site metastasis to occur, certain intrinsic alterations coupled with the extrinsic release of TME factors must occur. In a metastasis model, TLR2 and MyD88 knockouts in bone-marrow-derived macrophages (BMDM) significantly decreased IL-6 expression, a BMDM activation marker relative to other TLRs and adaptor protein TRIF (22). Furthermore, an *in vivo* murine model showed a significant reduction in macrophage infiltration and inflammatory cytokine expression in TLR2 knockout mice compared to wild type (WT), revealing the necessity of TLR2 recognition of a cancer cell ligand for promotion of metastatic growth (22). Alternatively, in mast cells TLR2 agonists altered the TME by producing cytokines and chemokines and by recruiting leukocytes (50, 51). This unique and selective TLR2 activation in mast cells restored the anti-tumor potential hence inhibiting lung carcinoma growth (32). As such, the TLR1:TLR2 complex has favorably been targeted as a promising biomarker because it inhibits lung tumor growth and decreases m-MDSCs (23). Both TLR1 and TLR2 have good prognoses for lung cancer and higher expression of these proteins may emerge as useful biomarkers in predicting the outcome or progression of lung cancer (43).

### TLR3 in lung cancer

Like TLR2, TLR3 also has conflicting roles in lung cancer as either pro- or anti-tumor receptors (52). In lung epithelial cells, TLR3 senses tumoral exosomal RNA as a critical step for initiation of neutrophil recruitment and lung metastatic niche formation (24). TLR3 has also been shown to recognize double-stranded RNA from lung tumor cells that activate the endothelial SLIT2 gene to drive metastatic growth forward (25). The direct activation of TLR3 by primary tumoral exosomal RNA induces chemokine secretion in lung epithelial cells (**Table 1**), and a direct correlation between reduced lung metastasis and TLR3 deficiency in mice has been established (24). Furthermore, this study highlighted TLR3s role in utilizing neutrophil recruitment to promote the lung pre-metastatic niche through activation by tumor-derived exosomal RNAs. On the flip side, in NSCLC, TLR3 has been observed *in vitro* to induce apoptosis of tumor cells and to activate lung DCs to elicit positive immune responses (33). The prognostic value of TLR3 is also opposing and dependent upon its expression on tumor versus immune cells. TLR3 expression on tumor cells elicited favorable NSCLC outcomes during early stages, whereas TLR3 expression on immune cells, primarily macrophages, elicited poor prognosis in patients’ survival (53). Thus, further research is needed to elucidate the role of TLR3 in tumor and immune cells and its dominant role in TME.

### TLR4 in lung cancer

TLR4 is one of the most widely studied receptors within the TLR family, with several synthetic agonists already approved as adjuvants in vaccines against immunogenic targets (54). TLR4 is seen at high levels of lung cancer tissue compared to non-lung cancer tissues (30). TLR4 is specifically shown to help lung cancer cells escape the immune system through the release of immunosuppressive cytokines like transforming growth factor beta (TGF-β), VEGF, and IL-8, while also increasing resistance to proapoptotic factors like tumor necrosis factor-alpha (TNF-α) (26). Interestingly, activation of TLR4 by lipopolysaccharide (LPS) upregulates programmed death ligand 1 (PD-L1), which is favorable for tumor cells in driving T cell exhaustion, thus contributing to immune escape (55). TLR4 has also been shown to positively correlate with malignancy (30). The association between TLR2 and TLR4 has been shown to correspond with a higher risk of several cancer types (56). TLR2 and TLR4 in a double knockout metastatic lung cancer model have been shown to influence the ability of the tumor to proliferate and grow, as well as to decrease the number of neutrophils associated with the tumor (57). Alternatively, TLR4 can exert potential anti-tumor activity in NSCLC, with calreticulin (CALR) serving as an antigen characteristically associated with cell death from immune cells (34) Additionally, activation of the CALR-TLR4-MyD88 signaling pathway promotes migration and maturation of DCs, a key step in tumor regression (34).

### TLR5 in lung cancer

The effects of TLR5 in lung cancer have mostly been associated with anti-tumor activity, with some indications of pro-tumor effects reported in other cancer types (58). Prognostic evaluations of NSCLC patients suggest a positive association between high TLR5 expression and survival (36). Additionally, TLR5 signaling increases upon flagellin treatment of NSCLC cells and can inhibit proliferation, migration, and invasion, establishing a correlation between TLR5 recognizing flagella of incoming bacteria into the lung (36). In A549 lung cancer cells, inhibition of a flagellin-derived agonist delayed tumor growth through the TLR5 and MyD88-dependent pathway and even suppressed cell viability (59). Flagellin treatment leading to TLR5 activation has also shown promising roles in macrophage recruitment in damaged or infected lung tissue and is proposed to afford protection against the host’s immune infection response (60).

### TLR7 and TLR8 in lung cancer

TLR7 and TLR8 have similar structures and can both recognize viral single-stranded RNA. Differences between them reside in their binding domains which exhibit unique specificities (39, 61). TLR7 is expressed by pDCs and found to be present in both B cells and myeloid cells. Conversely, TLR8 is absent in pDCs and B cells but highly expressed in myeloid cells (62). TLR7/8 may be regarded as a more advanced target pathway using synthetic agonists like resiquimod (R848). Specifically, R848 optimizes the host innate and adaptive immunity by recruiting and increasing DCs, NKs, and T cells, while reducing TME regulatory T cells (37, 63). In combination, R848 and nanoemulsion (NE) have shown significant anti-tumor effects in lung cancer models through tumoral T cell activation and lessening of T cell exhaustion (64). Alone, TLR7 can resolve inflammation and inhibit angiogenesis and survival in NSCLC (39). TLR7 is highly expressed in primary NSCLC tumor cells and affords resistance to chemotherapeutic agents (63, 65). Both TLR7 and TLR8 stimulation activate the NF-κB pathway, thought to improve cell survival, inflammation, and chemoresistance in primary lung cancer cells (28, 65). While the anti-tumor roles of these TLRs can be advantageous in drug and immunotherapy development, their utility may be compromised by their lesser understood pro-tumorigenic actions.

### TLR9 in lung cancer

TLR9 is highly expressed at the mRNA and protein levels in lung cancer (30). TLR9 can promote metastasis, with evidence that the agonist CpG ODN promotes and enhances tumor progression and proliferation (31, 66). NSCLC patients without metastasis have lower mitochondrial DNA (mtDNA) and lower TLR9 expression compared to those with metastasis; therefore, TLR9 and mtDNA can serve as potential biomarkers for lung cancer progression and metastasis (67). In contrast, clinical developments are underway utilizing TLR9 agonists, like CpG-A ODN and CpG-B ODN to activate pDCs and B cells, respectively, for the treatment of lung cancer (68, 69). The three CpG motif classes each induce different interferon-alpha levels that are utilized for stimulating different immune cell components like pDC maturation and NK cell activation (68). Furthermore, TLR9 agonists have shown some utility in combating checkpoint inhibitor blockades like PD-1 by priming T-cell responses against lung tumors (70).

## Toll-like receptors and lung cancer immunity

### A trend in lung cancer treatments

Targeted molecular therapies are often used in lung cancer patients who do not qualify for platinum-based doublet therapies or whose risk for treatment-associated toxicity is unacceptably high (71, 72). This has resulted in a new trend in lung cancer treatment that is based on immunotherapy. As noted earlier, current lung cancer immunotherapies continue to face challenges due to limited effectiveness and resistance.

### Current lung cancer immunotherapies

Two hallmarks of lung cancer diagnosis and treatment include the use of genomic technologies to profile tumors and the ability to identify unique predictive molecular targets that together can identify significant differences amongst tumors and their TME (73). Immune evasion by tumor cells is largely targeted in current treatments, and immunosuppressive factors like PD-L1/2 continue to be exploited (73). Immunomodulatory therapies readily focus on the interactions between PD-L1 on tumor cells and its receptor PD-1 on T cells because the binding of PD-1 to the PD-L1 breaks the activation of T cell anti-tumor function. Several immune checkpoint inhibitors such as nivolumab and pembrolizumab (two PD-1 blocking antibodies) have been approved by the FDA for use in lung cancer treatment, but only a small portion of lung cancer patients responds to these immune checkpoint inhibitors (38, 74). Some patients respond well at the beginning of treatment but fail to respond after 2-4 weeks due to T cell exhaustion. Some anti-PD-L1 antibodies like MPDL3280A are being used to prevent T cell exhaustion from occurring (38). However, the efficacy of these current immunotherapies needs to be improved.

TLRs play important roles in cancer immunity based on their ability to induce DC maturation, macrophage phenotypic modulation, enhancement of the B cell response, activation of NK cells, increased effector T cell activity, and promotion of suppressive regulatory T cell functions (75). Their anti-tumor properties have led to the consideration of TLR agonists for use in cancer immunotherapy (**Table 2**). In the 1800’s, the TLR4 ligand LPS was the first reported agonist used in an attempt to reduce tumor growth, yet today, we know that bacterial pathogens can promote lung cancer growth and metastasis (87). However, the use of TLR agonists in lung cancer remains controversial due to the poorly understood pro-tumorigenic properties of various TLRs. The underlying mechanisms responsible for the variable response need to be further investigated. Recently, the use of a TLR9 class A CpG agonist, Vidutolimod, was found to be effective in patients with resistance to PD-1 blockade (88). Furthermore, different types of CpG ODN like DV281 (Class C) and TLR7/8 agonists are being used in combination with immune checkpoint inhibitors like an anti-PD-1 antibody to induce immune cytokine and chemokine responses during lung cancer immunotherapy (**Table 3**) (40).

**Table 2:** Use of TLR agonists for lung cancer treatment.

TLRs	TLR Agonists	Experimental Model/Clinical Trials	References
TLR2	Pam2CSK4	Mouse	(76)
TLR2	CADI-05	Combined with chemotherapy in trial patients	(77)
TLR2/4	Polysaccharide	Lewis Lung Cancer (LLC)-bearing C57/BL6J mice and human NSCLC H460-bearing nude mice.	(78)
TLR3	Poly I:C	LLC/2 tumor-bearing mice	(79)
TLR4	Cucurbitacin B (CuB),	Mouse model (Cub, extracted from muskmelon pedicel, is a natural bioactive product)	(35)
TLR4	E. coli	C57BL/6 mice, promote metastasis	(80)
TLR4	L6H21 (an MD2 inhibitor)	Prevents lung metastasis in CT26 mouse model	(81)
TLR5	CBLB502	Nude mice (enhances radiosensitivity)	(59)
TLR7	TQ-A3334	Phase II Trial	(82)
TLR7	BNT411	Phase II Trial	(83)
TLR7/8	R848	C57BL/6 mice	(37)
TLR7/8	BDB001	Phase II trial in patients with PD-(L)1 refractory solid tumors, including NSCLC	(84)
TLR9	MGN1703	Phase II Trial	(27)
TLR9	IMO-2055	Combined with chemotherapy in Phase II trial	(85)
TLR9	PF-3512676	Combined with chemotherapy in Phase III trial (*last updated in 2015, but no study results are posted on clinicaltrials.gov)	(86)

**Table 3:** Combination of TLR agonists with checkpoint inhibitors for lung cancer treatment.

TLRs	TLR agonists	Treatment Models/clinic trials	References
TLR7/8	R848	R848 + anti-PD1 Ab, orthotopic models	(64)
TLR9	DV281 (ODN-C)	Inhaled aerosolized DV281 with anti-PD1 antibody in mice and primates	(40)
TLR9	Vidutolimod (ODN-A)	Treat patients with resistance to PD-1 blockade (clinic Phase Ib trial)	(88)

## Perspectives

Although research on the role of TLRs in cancer has been a hot-research area for many years, important questions remain to be addressed.

### Are infection-associated cancers underestimated?

It is well known that approximately 10-20% of cancer is associated with bacterial infections. In some cancers, specific infections are the main cause of diseases, such as HBV (Hepatitis B) for liver cancer and HPV (Human Papillomavirus) for cervical cancer. It is not surprising that TLRs recognize these viruses and play a crucial role in pathogen-associated oncogenesis. A recent study reported that bacteria-associated cancers are much higher than 20%, and that bacterial footprints like LPS can be detected in about 60-70% of some cancers (89). Thus, the availability of better-designed antibodies directed at bacterial-associated patterns along with improved detection methods, may make it possible to identify cancer pathogens. If we identify a pathogen as the main cause of a specific type of cancer, better strategies to prevent cancer initiation may be possible along with improved modalities for cancer treatment. Additionally, some viral infections such as HPV and HIV infections might also be associated with lung cancer. As mentioned above, HPV is a well-known causative agent for cervical cancer, but HPV can also infect other organs. Previously HPV was found to infect the esophagus and induce esophageal cancer (90). Alternatively, the validity of reports also indicates an association between HPV and lung cancer (91). The exact mechanism needs to be further investigated, but one *in vitro* study showed that the TLR3 signaling pathway might play a role in HPV-inducing lung cancer (92). Additionally, a different intriguing study shows that HIV-infected cells can release exosomes with special RNA recognized by TLR3 for promoting cancer cell proliferation (93). Many reports show that HIV infection is associated with lung cancer (94–96), but most people assume that this link is indirect (97, 98). However, it is worth determining the exact mechanism of how HIV-infected patients dramatically increase the risk of developing lung cancer. The lung is highly susceptible to pathogen exposure; therefore, the connection between bacterial and viral infections as well as mycotic infections and lung cancers should continue to be carefully investigated.

### Are there novel endogenous ligands of TLRs in cancers yet to be identified?

TLRs recognize not only pathogens but also endogenous danger molecules, particularly those derived from cancer cells. Recent structural analyses have identified novel binding amongst TLRs-PAMPs (99, 100) However, to date, the endogenous ligands for most TLRs have not been identified and TLRs-DAMPs binding modules remain poorly understood. The identification of endogenous TLRs ligands in cancer is incredibly important not only for a better understanding of tumor development, but also for the development of novel anti-tumor drugs.

### Can TLR activation regulate PD-1/PD-L1 and CTLA-4?

We posit that TLRs are ideal targets for therapeutic development. Some TLRs can induce anti-tumor activity, while others can regulate adaptive immunity. The resistance of cancer patients to immunotherapy has been associated with an immunologically “cold” TME where immune-killing activity is repressed (101). The activation of select TLRs can modulate the TME from an immunologically cold to an immune hot environment that is poised to improve the body’s own ability to fight the disease. Indeed, several clinical trials have used a combination treatment of TLR agonists and checkpoint inhibitors with promising results (64, 88). Additionally, combination therapies involving TLR7 and TLR9 agonists with PD-1 blockades have increased the proportion of M1 to M2 tumor-associated macrophages and induced infiltration of tumor-specific IFN-γ-producing CD8+ T cells to elicit tumor-specific adaptive immune responses and hence tumor suppression (102). However, the mechanism of how TLRs regulate the expression of PD-1, PD-L1, and CTLA-4 in various cell types remains to be investigated (102). Interestingly, a recent study has shown ligands of TLR1/2, TLR7, and TLR9 capable of downregulating PD-1 expression on CD8+ T cells through the release of IL12 by APCs (103). TLR7 agonists in combination with checkpoint inhibitors targeting PD-1 and CTLA-4 have been shown to be safe and effective in immunotherapy-resistant tumor models to promote more long-term immune responses (104). However, combination therapy has opposingly been reported to inhibit antitumor activity upon PD-1 blockade diminishing CpG-ODNs antitumor activity on macrophages to promote tumor growth (105). Additional mechanistic insights will lead to the elucidation of more effective regimens to target both innate and adaptive immunity against cancer and unravel the molecular determinants of the TLR response in tumor cells.

## Conclusions

TLRs are now being targeted for drug development and immunotherapy discovery because of promising innate immune activation. The usefulness of this approach is limited at this time due to conflicting pro- and anti-tumor activities of TLR receptors. The opposing effects of TLRs appear to be cell type- and microenvironment-specific, but the molecular determinants of these biological responses are not yet understood. Encouraging results from clinical studies using TLRs alone or in combination with other checkpoint inhibitors are beginning to appear. Therefore, there is a need to expand this field of research by exploring TLRs and their corresponding agonists and antagonists as promising tools in immunotherapy discovery and drug development.

## Author contributions

DZ and BH designed and outlined this review article; BH performed literature search and wrote the draft of the manuscript. All authors edited and approved the final manuscript.

## Funding

This study was partially supported by Texas A&M University, a grant from the Cancer Prevention & Research Institute of Texas (CPRIT, DP190020), and a NIH grant (R21CA176698).

## Acknowledgments

We appreciate the help from all our contributing authors in writing this review. We also want to acknowledge Texas A&M University for providing access to journals and the use of BioRender.

## Conflict of interest

The authors declare that the research was conducted in the absence of any commercial or financial relationships that could be construed as a potential conflict of interest.

## Publisher’s note

All claims expressed in this article are solely those of the authors and do not necessarily represent those of their affiliated organizations, or those of the publisher, the editors and the reviewers. Any product that may be evaluated in this article, or claim that may be made by its manufacturer, is not guaranteed or endorsed by the publisher.
